# Validation of the chinese version of the oral health impact profile for TMDs (OHIP- TMDs-C)

**DOI:** 10.4317/medoral.20243

**Published:** 2014-12-05

**Authors:** Song-lin He, Jin-Hua Wang

**Affiliations:** 1 Chongqing key Laboratory of Oral Diseases and Biomedical Sciences; Department of Pediatric Dentistry, Stomatological Hospital of Chongqing Medical University, Chongqing Medical University, Chongqing, China

## Abstract

Objectives: The aim of this study was to evaluate the reliability and validity of the the Chinese version of the Oral Health Impact Profile for TMDs (OHIP-TMDs-C). Study Design: The OHIP-TMDs was initially translated and cross-culturally adapted to Chinese following international guidelines; then subsequently validated for the psychometric characteristics of reliability and validity. In total, 156 participants with temporomandibular disorders (TMDs) were recruited to complete the questionnaire. The reliability of the OHIP-TMDs-C was evaluated using internal consistency and test-retest methods. The validity of the OHIP-TMDs-C was analysed by construct validity and convergent validity. Construct validity was determined based on factor analysis, and convergent validity by analyzing the correlation between OHIP-TMDs-C subscale scores and the global rating of oral health question. Results: Cronbach’s alpha value (internal reliability) for the total OHIP-TMDs-C score was 0.917 and the intraclass correlation coefficient (ICC) value (test–retest reliability) was 0.899. Construct validity was determined by factor analysis, extracting five factors, accounting for 78.6% of the variance. All items had factor loadings above 0.40. In terms of convergent validity, the OHIP-TMDs-C subscale was significant correlated to the global oral health rating. Conclusions: The results suggest that the OHIP-TMDs-C has good reliability and validity and thus may be used as a valuable instrument for patients with TMDs in China.

** Key words:**Validation, TMDs, quality of life, questionnaire.

## Introduction

Temporomandibular disorders (TMDs) are significant problems, not only for the individual who suffers from the condition but also for society that must bear the high economic cost of treatment and loss in productivity (1). Over the years, different theories of etiology and different emphases on the causative factors for the various signs and symptoms of TMDs have been proposed in the literature (2). It is a highly prevalent oral condition, affecting 14.9% to 17.9% of Chinese people (3,4). This oral disorder can significantly affect patients with clinical manifestations including severe daily orofacial, neck, and head pain, sleep dysfunction, and depression. In addition, functional activities that require optimal jaw mobility, such as eating, chewing, biting, kissing, and speaking, are impaired (5-7). Therefore, there is a need to comprehensively assess the impact of TMDs on patients’ daily living. Oral health-related quality of life (OHRQoL) is a multidimensional construct quantifying the extent to which oral disorders affect functioning, psycho social well being, sense of self, expectations and satisfaction with care. It has important implications for the clinical practice of dentistry and dental research. A number of systematic reviews of TMDs have gone on to suggest OHRQoL as an important outcome in treatment of TMDs (8,9).

Commonly used instruments to assess OHRQoL include the Oral Health Impact Profile (OHIP) and the Oral Impacts on Daily Performance (OIDP) (10,11). These instruments are large, however, and might include many redundant items, reflecting their generic nature. This would result in a reduction of its evaluative ability (12). They may be less useful for the assessment of the burden of specific oral disorders (e.g. dental aesthetics, oral mucosal diseases, dry mouth) on OHRQoL (13-15). Such generic measures can be too broad to accurately assess the links between specific oral conditions and OHRQoL. Durham and colleagues found that the particular impacts of TMDs were not fully captured through OHIP-49 (16). Several researchers suggested choosing a condition-specific instrument to assess its effect on people (17).

A Oral Health Impact Profile for TMDs (OHIP-TMDs) that assesses aspects of OHRQoL related to TMDs was recently proposed by Durham and colleagues (16). This condition-specific measure showed good psychometric properties in the UK. OHIP-TMDs could be used to evaluate negative impacts specifically related to TMDs, and aid in the development of effective interventions and health policies for TMDs. Yet this questionnaire cannot be directly used in non-English-speaking countries. Because of variations in social and, economic structures, culture and language, rigorous psychometric evaluation must be accomplished before it can be used in other areas. Therefore, this study aimed to translate the OHIP-TMDs into Chinese, to evaluate its cross-cultural adaptation, and to test its reliability and validity among Chinese people.

## Material and Methods

- Participants

A consecutive sample of 156 participants aged 18 years or older was recruited from the Affiliated Hospital of Stomatology, Chongqing Medical University. To be included, all participants had to undergo a standardized history taking and examination and, on the basis of this, meet the Research Diagnostic Criteria for Temporomandibular Disorders (RDC/TMD-Axis I) (18,19). In addition, they had to have been symptomatic for TMDs for longer than 6 months. Participants who have teeth pain, a history of psychiatric illness, a systemic disease, or unable to understand the OHIP- TMDs-C questions were excluded from the study.

Sample size calculation was based on the patient to item ratio of (5-10): 1 and was used in our previous validation study (20). A minimum of 110 patients were required because the questionnaire contained 22 items. Finally, 156 patients were selected to complete the OHIP-TMDs-C. Additionally, the result of the Kaiser-Meyer-Olkin (KMO) was 0.850, which exceeded the recommended value of 0.60 to proceed with the exploratory factor analysis. The result also indicated the adequacy of the sample size. A detailed explanation was provided before the patients filled out the OHIP-TMDs-C. The questionnaire was completed inside a waiting room. The participants could consult the research assistants at any time if they had any questions.

A positive ethical approval was obtained from the Ethics Committee of Chongqing Medical University, and all participants signed an informed consent form.

- The OHIP-TMDs

OHIP-TMDs is an English-language OHRQoL instrument, developed by the Newcastle University, UK. It consists of 22 items ( two items were newly added in the OHIP-TMDs: Have you had difficulties in opening and closing your mouth? and Have you felt speech was painful because of problems with your teeth, mouth, dentures or jaws?) grouped into seven domains to describe the functional limitation (items 1-2), physical pain (items 3-7), psychological discomfort (items 8-11), physical disability (items 12-13), psychological disability (items 14-18), social disability (items 19,20) and handicap (items 21,22). The response is a five-point Likert format: Never, Hardly ever, Occasionally, Fairly often, Very often (equivalent to scores of 0-4). In order to examine convergent validity, an extra global oral health question (“In general, how would you rate your temporomandibular joint status”) was added at the end of instrument. The possible responses to this question were “very good” “good” “fair” “poor” “very poor”, and scores of 1-5 respectively were assigned to the aforementioned responses.

- Translation and cross-cultural adaptation

The OHIP-TMDs was translated into Chinese using the forward–backward process proposed by Guillemin *et al*. (21). The process included several major steps:

1) Two independent translators first translated the OHIP-TMDs from English to Chinese (based on the Mandarin Chinese). Both translators were fluent in English and Chinese and had background knowledge of dentistry.

2) Then, the two independent versions were back-translated from Chinese to English by a professional English teacher and two bilingual dental specialists, none of whom knew the original questionnaire.

3) The translated and back-translated versions were compared and discussed by an expert panel consisting of two dental specialists with extensive knowledge of OHRQoL assessment who were fluent in both English and Chinese. A preliminary Chinese OHIP-TMDs version was then produced.

4) The preliminary Chinese OHIP-TMDs was pilot tested on a convenience sample of 20 participants.

5) After the test, emerging problems were discussed. The Chinese version was considered final when there were no substantial differences.

- Statistical Analysis

Reliability: Two types of reliability were adopted to assess the reliability of the OHIP-TMDs-C. Internal consistency was evaluated by calculating Cronbach’s alpha and test-retest reliability was determined via Intraclass Correlation Coefficients (ICC) using data from the 30 participants who completed OHIP-TMDs-C again after a 2-week interval. Cronbach’s alpha of 0.70 or greater is considered acceptable for comparisons between groups. Descriptors for ICC denoting poor to fair, moderate, good and excellent agreement correspond to scores of <0.40, 0.41-0.60, 0.61-0.80 and > 0.80 respectively (22).

Validity was assessed as construct and convergent validity. Construct validity was determined using exploratory factor analysis (varimax rotation). However, a Bartlett’s test of sphericity coefficient and Kaiser-Meyer-Olkin (KMO) test must be firstly conducted to determine whether there are sufficient significant correlations among items to carry out this analysis (23). An eigenvalue of over 1 were regarded as a criterion for factor extraction. Factor loadings greater than 0.40 were considered significant. Finally, convergent validity was tested through investigating the correlation between OHIP-TMDs-C sub scale scores and the global oral health question. The correlation values is considered to indicate poor correlation when < 0.20, to indicate fair correlation when 021-0.40, to signify good correlation when 0.41-0.60, to indicate very good correlation when 0.61-0.80, and to indicate excellent correlation when > 0.81 (24). Statistical analyses were conducted by SPSS 20.0 (SPSS, Chicago, IL, USA).

## Results

- Sample characteristics

A total of 156 participants were recruited from a university-affiliated clinic for this study. All the OHIP-TMDs-C questionnaires were completed fully. The mean age of the participants was 37.7 ± 15.3 years (range 18-83), and the 65.4% were female.  Table 1 presents the characteristics of participants. The mean scores, corrected item- total correlations and factor analysis results for the OHIP-TMDs-C are presented in  Table 2.

**Table 1:**
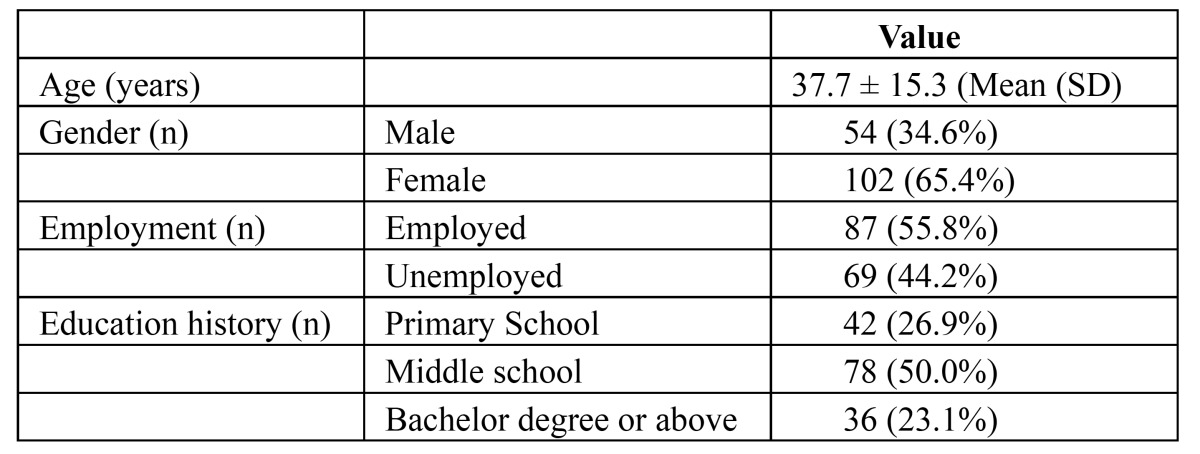
Characteristics of participants (n = 156).

**Table 2:**
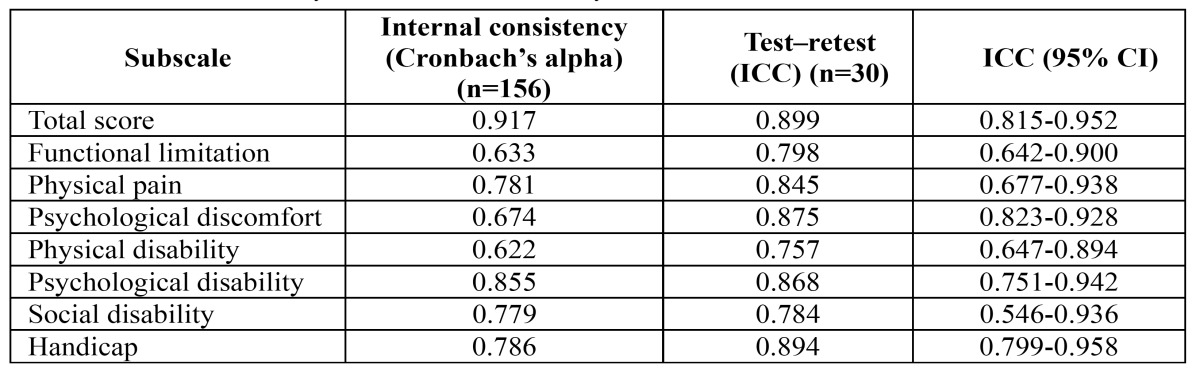
Internal consistency and test–retest reliability of the OHIP-TMDs-C.

- Reliability

 Table 3 shows the internal consistency of the multi-item scales. Cronbach’s alpha for the total score of OHIP-TMDs-C was 0.917 and values for the sub scales ranged from 0.633 for ‘functional limitation’ to 0.855 for ‘psychological disability’. All sub scales exceeded the minimum reliability standard of 0.70, except the functional limitation sub scale, whose value of 0.633 nearly reached the threshold. The corrected item-total correlations ranged from 0.357 (item 3 and item 12) to 0.739 (item 22). All items reach the recommended minimum correlation of 0.20.

**Table 3:**
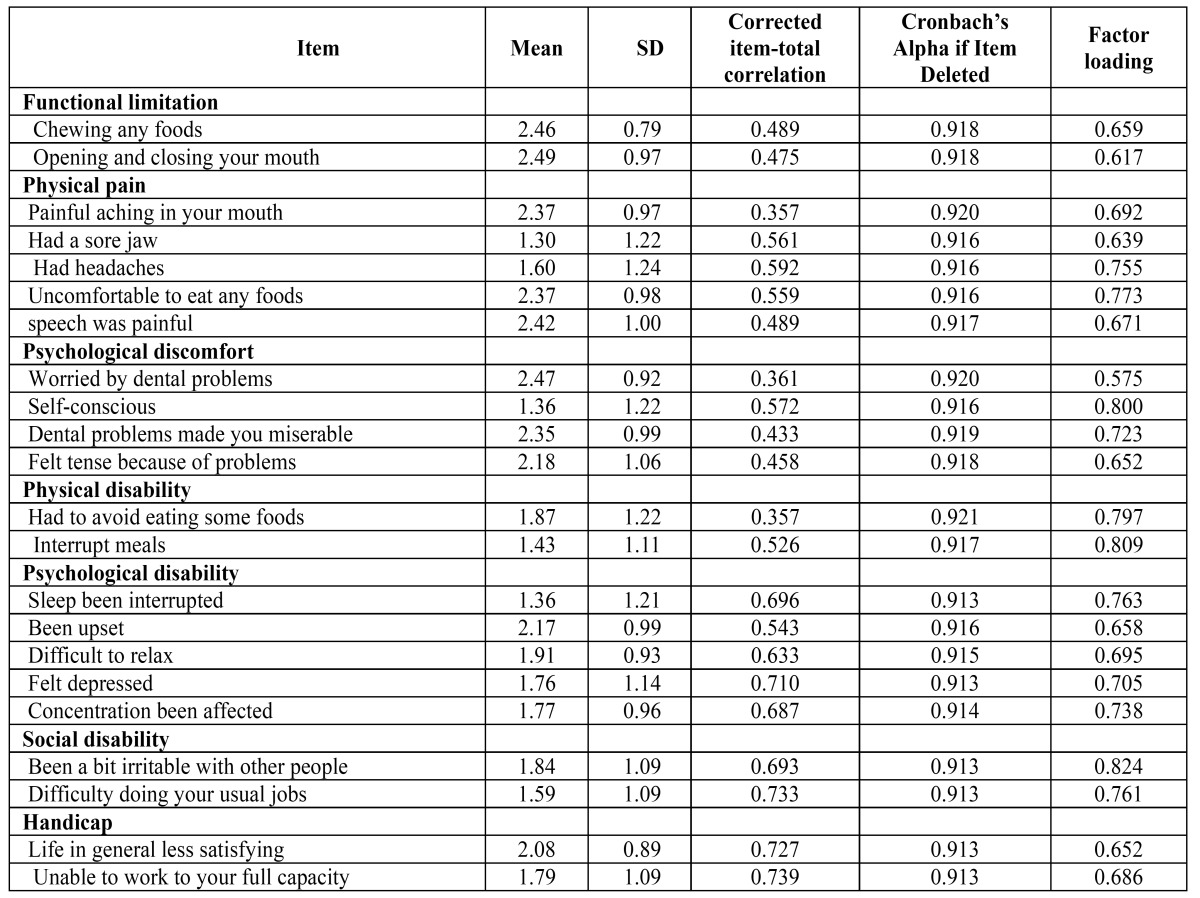
Range, mean scores, corrected item-total correlations and factor analysis results for the OHIP-TMDs-C.

Test–retest reliability was calculated for the 30 participants who repeated the test after two-weeks. The 95% confidence intervals of the means were computed. ICCs for the total score of OHIP-TMDs-C was 0.899 and values for the sub scales ranged from 0.757 (95% CI = 0.647-0.894) to 0.894 (95% CI =0.799-0.958), indicating an excellent agreement. Overall, these results suggested good reliability for the OHIP-TMDs-C.

- Validity

Construct validity was assessed through factor analysis. The result of the KMO test was 0.850 and Bartlett’s test of sphericity was 2287.1 (df = 231, *P* < 0.001) demonstrating sufficient significant correlations to perform factor analysis. The results of the factor analysis for the sub scales are presented in  Table 3. All items had factor loadings above 0.40. Factor analysis extracted five factors which together accounted for 78.6% of the variance. Total and sub scale scores of the OHIP-TMDs-C had significant correlations with global oral health status (rs ranged from 0.290 to 0.548), thus indicating fair to good convergent validity ( Table 4).

**Table 4:**
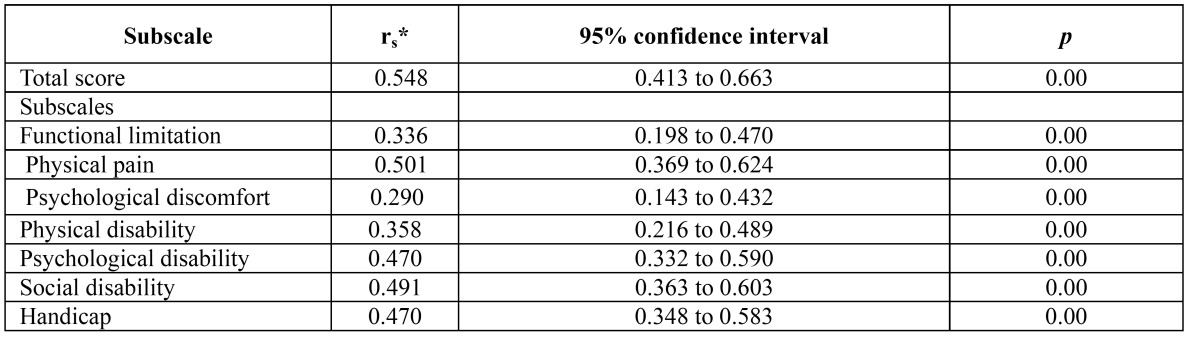
Convergent validity of the OHIP-TMDs-C: Correlations between subscale scores and global oral health rating.

## Discussion

To the best of our knowledge, this study provides the first introduction of the OHIP-TMDs in a non-English-speaking country and also constitutes the first evaluation of the psychometric properties of the OHIP-TMDs in a non-English-speaking country. In recent years, a large number of studies assessing OHRQoL have been conducted (13-15). Foreign instruments must be adapted before they can be used on speakers of different languages in other cultures. Therefore, we performed a cross-cultural adaptation of the original OHIP-TMDs and evaluated the reliability and validity of the OHIP-TMDs-C according to international standards. The results presented here demonstrate the utility of the OHIP-TMDs-C to measure the psychosocial impact of TMDs.

The translation of an existing instrument into another language is an important procedure in the cross-cultural adaptation of a quality of life instrument (25,26). In this study, cultural and conceptual equivalence were obtained via international guidelines for cross-cultural adaptation of psychometric measurements. The results demonstrated that linguistic and cultural equivalence of Chinese and English versions of OHIP-TMDs. China has the largest population in the world, and more than 870 million people speak Mandarin Chinese. This necessitates the development of a Chinese version of the OHIP-TMDs, which would have wide clinical and research applications.

Reliability is an important dimension of any patient-based outcome measure as it is essential to establish whether changes observed are due to the intervention and not to variations related to problems with the outcome instrument. There are two aspects that have to be considered when evaluating reliability: internal consistency and test-retest reliability. For internal consistency, Cronbach’s coefficient alpha to test reliability exceeded 0.70 for all measures except for the functional limitation sub scale. The corrected item-total correlations were all well above the recommended level of 0.2. These results demonstrate that the OHIP-TMDs-C has good internal consistency reliability.

To evaluate test-retest reliability, it is generally suggested that the time period between repeated administrations should be long enough to prevent recall but short enough to ensure that clinical changes have not occurred. No definitive time interval has been experimentally determined, but a period of one to two weeks is often considered appropriate (27). Test-retest reliability for the OHIP-TMDs-C and subscales demonstrated good to excellent agreement. These findings indicate that the OHIP-TMDs-C is a reliable and stable instrument for assessing the impacts of temporomandibular disorders.

Another important psychometric property of a questionnaire is its validity. Construct validity refers to the extent to which scores on a particular instrument relate to other measures in a manner that is consistent with the theoretically derived hypotheses on the concepts being measured (28). In addition, convergent validity investigates how closely the new scale relates to other measures of the same construct. In factor analysis, all items had factor loadings of >0.40, indicating that all items had strong relationship to their factors. Furthermore, significant correlations were observed between OHIP-TMDs-C scores and global oral health rating. Overall, the OHIP-TMDs-C showed fair to good convergent validity.

However, some limitations of the present study should be considered. First, the sensitivity and responsiveness of the OHIP-TMDs-C were not conducted, because this will require a longitudinal study. Additionally, all participants were recruited from the Affiliated Hospital of Stomatology; thus, the results cannot be extrapolated to general population of China. Further research of the OHIP-TMDs-C should be performed in a sample of general population to confirm the generalizability of the findings.

In summary, this study confirmed the reliability and validity of the OHIP-TMDs-C. It was appropriate to employ the questionnaire to assess the OHRQoL of patients in China who experience from temporomandibular disorders.

## Conclusions

The English OHIP-TMDs was successfully translated into Chinese and culturally adapted for use in mainland China. This study provided preliminary evidence concerning reliability and validity of the OHIP-TMDs-C. The results provide initial evidence that the OHIP-TMDs-C may be a valuable instrument for assessment of TMDs in China.
